# END-OF-LIFE RESOURCE AND PLACE OF DEATH BY COMMUNITY-BASED HOUSING TYPE

**DOI:** 10.1093/geroni/igae098.2789

**Published:** 2024-12-31

**Authors:** Sojung Park, BoRin Kim, Esther Shin, Jihye Baek, Byeongju Ryu

**Affiliations:** Washington University in St. Louis, St. Louis, Missouri, United States; University of New Hampshire, Durham, New Hampshire, United States; Illinois State University, Normal, Illinois, United States; Washington University in St. Louis, St. Louis, Missouri, United States; Washington University in St. Louis, St. Louis, Missouri, United States

## Abstract

Understanding the factors that influence where people die is crucial, as most individuals spend their last year of life at home. This study investigates the influence of end-of-life resources on the place of death in older adults, based on where they lived. Data came from twelve waves of the National Health and Aging Trend Study (2010~2022). We analyzed the unmet needs of end-of-life resources such as end-of-life care services, functional help, and social/family support, and the type of housing (senior housing vs. traditional home) based on income status. The sample included community dwellers who died during the study period and had information about end-of-life care-related supports (N= 2701). Multinominal regression analyses were used to investigate the influence of end-of-life resources and housing type on the place of death (home, hospital, nursing home, hospice facility, or other). We found individuals living in traditional homes, both low-income and non-low-income, tend to have low levels of end-of-life care services but receive high levels of social/family support and they are more likely to die at home. In contrast, individuals living in senior housing, particularly those in low-income models, tend to have high levels of unmet needs for functional help and social/family support and are more likely to die in a nursing home or other facilities. We discussed the unequal distribution of end-of-life resources based on residential type, and the implications of this on the apparent mismatch between unmet resources and the place of death, focusing on the potential role of senior housing.

